# Ileal Gastrointestinal Stromal Tumor (GIST) Presenting With a Liver Abscess: A Case Report and Review of the Literature

**DOI:** 10.7759/cureus.78172

**Published:** 2025-01-29

**Authors:** Mohammed K Aljasser, Ahmed M Alshammari, Nawwaf H Alharbi

**Affiliations:** 1 General Surgery, Buraidah Central Hospital, Buraidah, SAU

**Keywords:** gastro-intestinal stromal tumor, gastrointestinal stromal tumors (gists), liver abscess, liver abscess drainage, small intestinal tumor

## Abstract

Gastrointestinal stromal tumors (GISTs) are rare small intestinal tumors. Due to challenging diagnoses, the majority of patients typically report chronic problems. Early diagnosis and the start of appropriate treatment depend on high suspicion. We present a case of a 47-year-old male patient who first experienced fever, nausea, vomiting, and pain in the right upper quadrant. Radiologic examinations showed an intra-abdominal tumor containing oral contrast and a liver lesion, indicating gastrointestinal tract involvement. He had an ileal GIST that created a fistula between the tumor and the small intestine, complicated by a liver abscess. We surgically removed the tumor and intra-operatively drained the liver abscess. A liver lesion found in conjunction with a small intestine GIST should cause concern for both the possibility of infectious complications such as a pyogenic liver abscess and metastatic illness. The uncommon appearance of an ileal GIST with a tumor-intestinal fistula, accompanied by a liver abscess, is reviewed in this case report.

## Introduction

The most common mesenchymal tumors of the gastrointestinal tract are gastrointestinal stromal tumors (GISTs) [1,2]. According to Hirota and colleagues, most GISTs are identified on immunohistochemistry by the expression of CD117 (KIT), which distinguishes them from other spindle cell tumors [3]. The KIT oncogene in these cancers contains an activating mutation, which has greatly advanced our knowledge of the biology underlying this uncommon illness and, as a result, greatly improved treatment [3]. The stomach (60%) and small intestine (20-30%) are the most common anatomical sites where GISTs are diagnosed [1]. The colon, rectum, appendix, esophagus, mesentery, omentum, or retroperitoneum are less common locations for them [1,2]. Abdominal pain, early satiety, abdominal distension, gastrointestinal bleeding, and a growing mass are some of the vague symptoms that many GISTs come with [1,2]. Though uncommon, perforation and obstruction are more frequent in small intestine GISTs than stomach GISTs [4]. Less frequently observed presentations encompass tumor perforation, GIST-related fistulas, and intra-abdominal abscesses [5-8].

## Case presentation

A 47-year-old Sudanese male farmer presented to the emergency department with a history of fever for one month on/off. Associated symptoms were abdominal pain, constipation, nausea, and vomiting that started suddenly in addition to loss of appetite and weight loss with no trauma history. There was a history of multiple ER visits for the same complaint receiving conservative management such as analgesia, intravenous fluids, and antibiotics for temporary relief of symptoms without improvement. The patient's medical records showed a diagnosis of diabetes mellitus on oral medication, gliclazide, with no surgical history. He denied any history of tobacco, alcohol, and recreational drug use. Upon clinical assessment, the patient was normotensive, tachycardic, and had a 39°C temperature. The right upper quadrant, right lower quadrant, and suprapubic abdomen area showed tenderness upon physical examination. An upright X-ray of the chest and abdomen showed nothing unusual. Laboratory results indicated an elevation in the leukocyte count (white blood cell count was 15 on arrival). Other labs investigated random blood sugar, platelet count, liver enzymes, hemoglobin (HGB), sodium, potassium, magnesium, and calcium levels mentioned in Table 1. However, all coagulative profiles and venous blood gas sampling for pH and lactate were normal.

**Table 1:** Serum lab investigations. RBS: Random blood sugar

Laboratory test	Result	Reference range
WBCs	15 x 10^3^/μL	4.5-11 x 10^3^/μL
Hemoglobin (HGB)	10.5 g/dL	13.5-17.5 g/dL
Platelets (PLT)	644 x 10^3^/μL	150-450 x 10^3^/μL
Serum sodium	126 mmol/L	136-146 mmol/L
Serum potassium	3.20 mmol/L	3.5-5.1 mmol/L
Serum magnesium	0.60 mmol/L	0.73-1.06 mmol/L
ALT	75.4 U/L	3-50 U/L
AST	82.70 U/L	3-50 U/L
Total bilirubin	12.90 umol/L	5-21 umol/L
Direct bilirubin	3.80 umol/L	0-3.4 umol/L
Albumin	21.60 g/L	35-52 g/L
RBS	21.30 mmol/L	3.9-7.8 mmol/L

**Figure 1:**
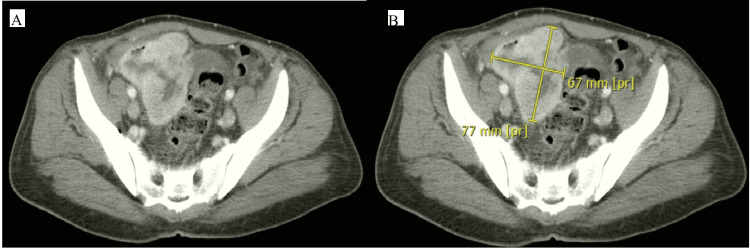
CT scan of the abdomen and pelvis with an intra-abdominal mass. The left image is without a marker, and the right image with a mark points to the mass, which is a pelvic intra-abdominal mass that measures about +/- 7.5x8x9 cm in maximal diameter.

**Figure 2:**
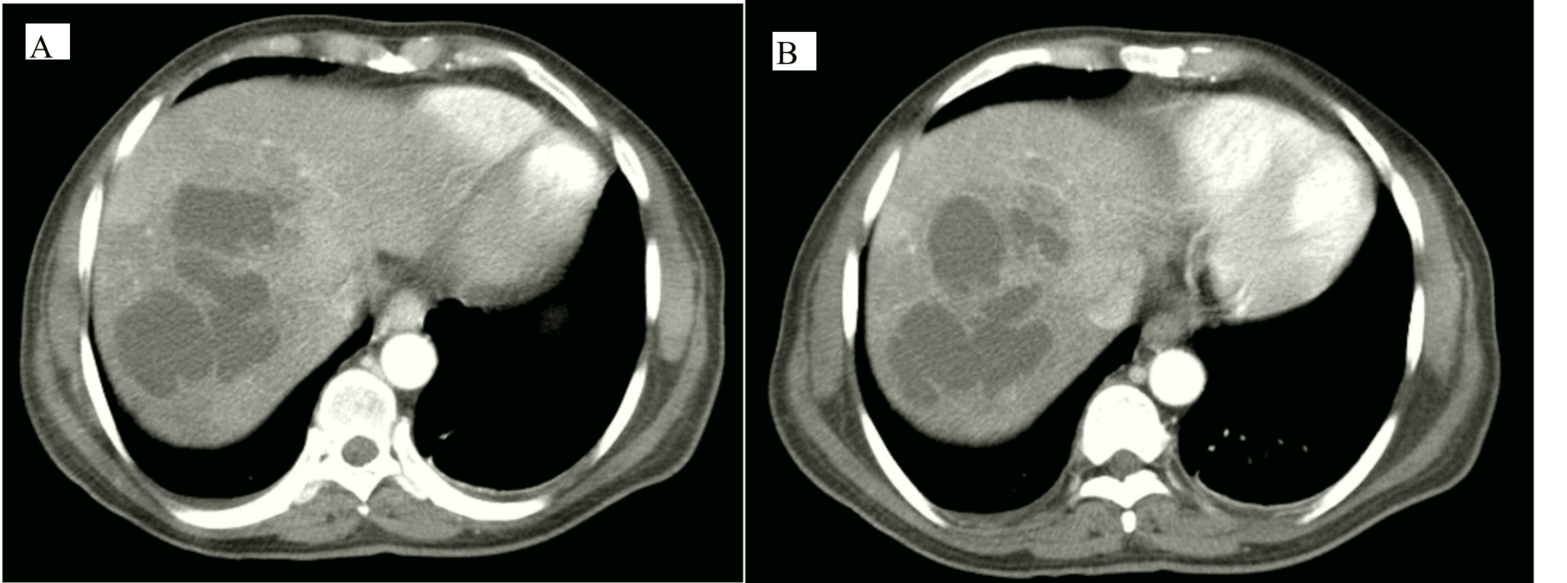
CT scan of the abdomen showed a liver lesion. Both images of axial CT with different levels show multiple loculated coalescent hepatic cystic lesions implicating the right hepatic lobe (segment VIII, VII) with a 13 cm CC diameter.

A CT scan revealed the right lower pelvis lobulated heterogeneous peripherally enhanced mass with a hypoechoic interior; the lesion, assumed to have originated from the pelvic ileal loops, has a maximal diameter in anteroposterior (AP), transverse (TR), and craniocaudal (CC) measures of about 7.5x8x9 cm, respectively. The lesion shows air-fluid levels with few scattered air locules extended beyond its wall, suggesting concealed perforation with mild pelvic free fluid (Figure 1). There were thickened, edematous, enhanced jejunal-ileal loops; the rest of the large bowel shows a loaded, constipated colon with thickened, enhanced parietal peritoneum. The visualized appendix measures about 8.8 mm in diameter and shows wall hyperenhancement (from the adjacent inflammatory process) and misty edematous congested mesentery. In addition, multiple loculated coalescent hepatic cystic lesions were seen implicating the right hepatic lobe (segment VIII, VII) with a 13 cm CC diameter, which is a hepatic abscess (Figure 2).

**Figure 3:**
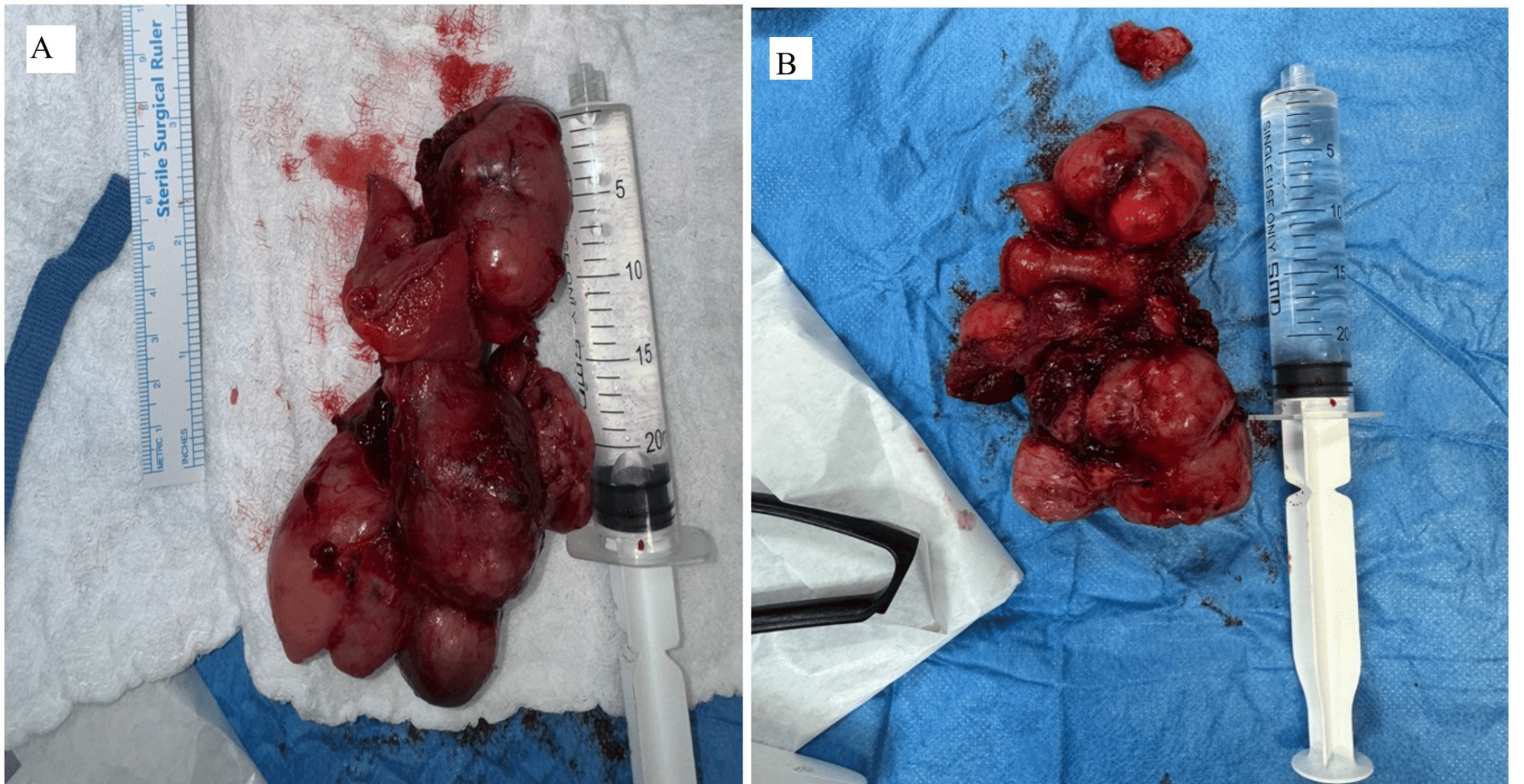
The part of the ileum containing the tumor mass with a size of around 9x6x3 cm was excised.

**Figure 4:**
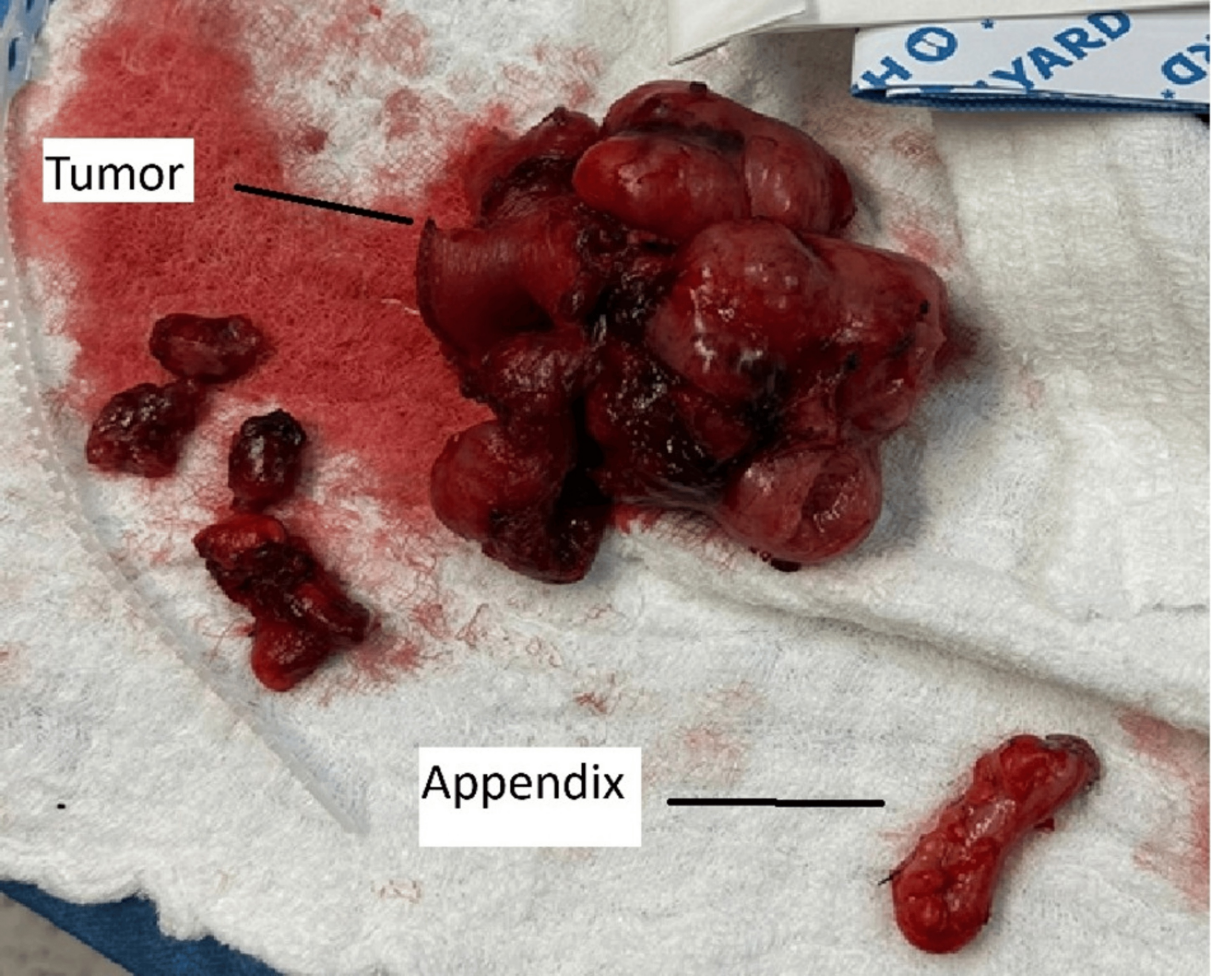
Excised tumor mass and the appendix.

After an extensive discussion with the patient, surgical exploration was planned. Surgery was performed by a general surgeon consultant with the assistance of specialists and a general surgery resident. At surgery, a large pelvic mass that was attached to two ileal segments was discovered. Adherent was the medial aspect of the sigmoid colon, mesocolon, and small bowel mesentery. Although there was considerable adhesion inflammation in the surrounding tissue, there was no evidence of enteric leakage in the intra-abdominal cavity. The mass was an exoteric, spherical, lobulated mass that originated in the ileum and was linked to the bladder wall, containing a sizable extraluminal component. There were no more suspicious abdominal lesions seen. A part of the ileum was excised, containing a mass approximately 10 cm from the ileocecal junction (Figures 3, 4). The ileum was anastomosed side to side using an EndoGIA stapler size 60 purple. Dissection of the mass encircling it, its release from the bladder, and its safe 2 cm margin of excision were done. However, the appendix was inflamed with multiple adhesions, so it was excised (Figure 4).

A gross examination of the liver revealed nothing abnormal, but aspirating pus from the right liver lobe after inserting a size 18 needle there. A chest tube was placed at the liver's right lobe, draining pus that was attached to a suction device. About 250 milliliters of pus emerged, and a sample was obtained for a microscopic examination and culture and sensitivity C/S. Two 16-sized drains were inserted, one in the pelvis at the anastomosis and the other in the right side subphrenic region.

Postoperatively, the patient was shifted to the surgical ward extubated, vitally stable, and afebrile. Day 1 post exploratory laparotomy abdominal drains: where the liver drain was 250 ml pus, the right subphrenic drain was minimal at 50 ml, and the left pelvic drain at the site of anastomosis was 35 ml serous. Drains were decreasing in amount gradually day by day with the improvement of the patient clinically, lab-wise, and radiologically. The patient was kept in the ward for around 32 days as the liver abscess was not accessible with interventional radiology, so conservative management with antibiotics was advised by infectious disease physicians until complete resolution. 

The final histopathology revealed a GIST spindle cell type, unifocal with a size of 9.0x6.0x3.0 cm, originating from the small intestine ileum, excluding the ileocecal valve, protruded through an opening of bowel wall measuring around 2.0x1.5 cm, with a mitotic rate of 12-15/5 mm² at high-power fields, histological grade G2, and high grade with necrosis seen at 5-10% (Figure 4). The histopathology from the appendix revealed mucosal acute nonspecific inflammation and focally hyperplastic mucosal epithelium that is tumor-free (Figure 4).

## Discussion

GISTs are the most prevalent gastrointestinal tract mesenchymal tumors, and it is believed that they originate from a common precursor cell of the Cajal interstitial cells [1,2]. Mutations that activate one of the receptor protein tyrosine kinases, such as PDGFRA or KIT (CD117), cause about 85% of these tumors [1]. About 10% of GISTs are wild-type tumors, which means they lack any observable mutations in PDGFRA or KIT [1]. Based on factors like size, mitotic index, anatomic location, and rupture existence, GISTs are classified as having a low, intermediate, or high risk of recurrence [1,2]. Establishing recurrence risk is essential for determining which individuals would benefit from adjuvant therapy. The Armed Forces Institute of Pathology (AFIP) criteria and the National Institutes of Health (NIH) criteria in the United States are the two most commonly used risk classification schemes [9-11]. Small bowel GISTs had disease-specific survival rates of 84.4, 71.2, and 54.2% after five, 10, and 20 years, respectively, making them the second most prevalent type of GIST in the digestive tract [12].

Management of GISTs is through complete resection with intact pseudo-capsule and negative microscopic margins [10]. The low incidence of lymph node metastases makes lymphadenectomy uncommon [10]. The standard therapy for inoperable, metastatic, or recurrent GISTs is imatinib mesylate, a selective inhibitor of a family of tyrosine kinase enzymes that includes KIT and PPDGFRA [9,10]. It has been successfully applied in both adjuvant and neoadjuvant settings [9,10]. For large, poorly positioned GISTs, preoperative imatinib is advised to facilitate organ-preserving surgery. Imatinib is advised for some intermediate- and high-risk GIST tumors in the adjuvant setting since it has been demonstrated to increase recurrence-free survival [9,10,11,13,14].

GIST's clinical presentation varies greatly depending on the size and location of the tumor. In general, symptoms are often ambiguous. Rare presentations are caused by intestinal obstruction, perforation, abscess, and the development of fistulas [4,7,13,15]. A case report of a patient from China in 2022 was presented with a history of frequent and urgent urination for two weeks. Radiologic studies revealed an ileal giant cystic GIST with atypical intratumoral abscess formation in the lower abdomen that was managed by complete surgical resection and adjuvant imatinib with follow-up for nine months with no recurrence of the tumor [13]. GISTs have the power to compromise the integrity of the gastrointestinal mucosa, creating an opening for colonizing bacteria to enter the bloodstream. According to earlier research, enteric bacteria enter the tumor cavity through the tumor-small intestinal fistula as a result of GISTs' propensity to cause mucosal ulceration or defects. This eventually leads to the development of an intratumoral abscess [5]. Liver abscesses can arise from infections in the portal vasculature, local bacterial spread from adjacent sites of infection inside the peritoneal cavity, or hematogenous dissemination of organisms in conjunction with systemic bacteremia [15].

Hepatic abscesses are often treated with drainage and intravenous antibiotics [16,17]. The link between pyogenic liver abscesses and GISTs has been documented in several earlier studies [16-20]. Metastatic illness is typically at the top of the differential when liver abnormalities and an intra-abdominal tumor are discovered. Pyogenic liver abscesses should be taken into consideration, particularly in patients with systemic symptoms, and antibiotic therapy should be started rapidly. Since his clinical picture indicated sepsis, the patient in this case was placed on intravenous antibiotics right away. Rather than a liver abscess, our first thought was that the patient had metastatic illness. The CT scan results were worrisome for a small perforation, which is why the surgical intervention was sought despite continuing concerns about distant disease. It was determined that the intra-abdominal mass was the cause of the patient's systemic symptoms and persistent infection, which required resection.

## Conclusions

We report the case of a large GIST located in the ileum diagnosed through histopathology after surgical excision with the complication of a liver abscess. This created a fistula between the tumor and the lumen of the small intestine, which allowed bacteria to move into the portal and systemic circulations and cause bacteremia and liver abscess. For uncommon presentations, a GIST should be ruled out before surgery for any intraperitoneal cystic lesions, particularly those that are thought to have gut origins. We have shared our clinical experience to help guide the management of similar cases.
